# Effects of Regular Taekwondo Intervention on Oxidative Stress Biomarkers and Myokines in Overweight and Obese Adolescents

**DOI:** 10.3390/ijerph17072505

**Published:** 2020-04-06

**Authors:** Hee-Tae Roh, Su-Youn Cho, Wi-Young So

**Affiliations:** 1 Department of Physical Education, College of Arts and Physical Education, Dong-A University, Busan 49315, Korea; dau0409@dau.ac.kr; 2 Department of Taekwondo, Youngsan University, Yangsan-si 50510, Korea; 3 Sports and Health Care Major, College of Humanities and Arts, Korea National University of Transportation, Chungju-si 27469, Korea

**Keywords:** adolescents, exercise training, myokine, obesity, oxidative stress, physical fitness, taekwondo

## Abstract

*Purpose*: Regular exercise can alleviate oxidative stress related to obesity and can induce secretion of myokines that are involved in the regulation of metabolic homeostasis. There are no studies examining changes in these variables as a result of Taekwondo training intervention. We aimed to investigate the effect of Taekwondo training on oxidative stress and myokine levels in overweight and obese adolescents. *Methods:* We randomly assigned 20 overweight and obese adolescents to control (control group; CG, n = 10) and experimental (experimental group; EG, n = 10) groups. The EG performed Taekwondo training five times a week for 16 weeks. Physical parameters (height, weight, body mass index (BMI)), physical fitness (maximal oxygen uptake (VO_2_max) (cardiorespiratory endurance), grip and leg strength (muscular strength), sit-and-reach (flexibility), Sargent jump (power), and stork stand test (balance)) were measured before and after intervention. We measured levels of serum oxidative stress markers (plasma malondialdehyde (MDA) and superoxide dismutase (SOD)) and myokines (serum interleukin-15 (IL-15), brain-derived neurotrophic factor (BDNF), irisin, and myostatin). *Results:* The weight and BMI in the EG after intervention were significantly lower and leg strength (muscular strength), sit-and-reach (flexibility), and Sargent jump (power) were significantly improved compared to those of the CG (*p <* 0.05). There were no significant interaction effects in terms of height, VO_2_ max, grip strength, or stork stand test (*p >* 0.05). The SOD and BDNF level after intervention were significantly higher in the EG after the intervention, whereas MDA and irisin levels were significantly lower than those of the CG (*p <* 0.05). There were no significant interaction effects in terms of serum IL-15 and myostatin levels (*p >* 0.05). *Conclusions:* Taekwondo training can reduce obesity and increase physical fitness with respect to muscular strength, flexibility, and power as well as alleviate oxidative stress and modulate myokine secretion in adolescents.

## 1. Introduction

Childhood and adolescent obesity have emerged as important public health problems worldwide [1,2]. The increase in prevalence of obesity in children and adolescents has occurred in Korea as well. According to the Korea National Health and Nutrition Examination Survey (KNHANES), the childhood and adolescent obesity rate was 5.8% in 1997, increasing to 7.6% and 11.5% in 2007 and 2018, respectively [3,4]. Obesity affects almost all body systems, including endocrine, gastrointestinal, pulmonary, cardiovascular, and musculoskeletal systems, inducing comorbidities such as type 2 diabetes mellitus, dyslipidemia, obstructive sleep apnea, and steatohepatitis [1]. Oxidative stress increased by obesity can exacerbate the severity of these diseases [5,6,7,8]. In particular, fat accumulation correlated with systemic oxidative stress, and increased fatty acids level increased oxidative stress through nicotinamide adenine dinucleotide phosphate (NADPH) oxidase activation [5,8]. Treatment with NADPH oxidase inhibitors decreased reactive oxygen species (ROS) production in adipose tissue and attenuated dysregulation of adipocytokines, improved diabetes, hyperlipidemia, and hepatic steatosis [5].

A bout of high intensity exercise induces excessive ROS production that is linked with lipid and peroxidation, having deleterious impact on cells and tissues [9]. However, regular exercise with proper intensity is an effective way to prevent and improve obesity by increasing the activation of antioxidant enzymes such as superoxide dismutase (SOD) and glutathione peroxidase (GPx), resulting in alleviation of oxidative stress [10,11,12,13]. In a study by Baghaiee et al., eight weeks of aerobic exercise significantly decreased serum levels of malondialdehyde (MDA) (an oxidative stress marker in obese subjects) and significantly increased SOD-2 gene expression [12]. Roh and So studied obese subjects (body mass index (BMI) < 25 kg/m^2^) and showed significantly higher serum ROS and lower serum SOD activities in obese than in nonobese subjects (BMI ≥ 25 kg/m^2^); eight weeks of aerobic exercise training significantly lowered BMI and percent body fat and gave rise to higher serum SOD activities in the obese subjects [13]. Exercise also ameliorates obesity and oxidative stress in adolescents. Li et al. conducted four weeks of exercise intervention and dietary restriction among 20 obese adolescents (age: 15.5 ± 2.1 years) and found significant decrease in BMI, lean body mass, body fat mass, and fat mass ratio as well as marked improvement of antioxidant enzyme levels such as SOD and GPx. However, this study, which examined changes in oxidative stress levels after exercise in obese adolescents, was relatively restricted in contrast to studies on obese adult subjects [14].

Adipose tissue in obese conditions secretes adipokines that contribute to chronic inflammatory environments, promoting pathological processes such as atherosclerosis and insulin resistance [15]. By contrast, exercise induces secretion of myokines that compensate for the harmful effects of these proinflammatory adipokines [15]. Myokines control autocrine function in muscle metabolism and paracrine/endocrine regulatory function in distant organs and tissues such as bone, adipose tissue, brain, and liver [16]. The currently known major myokines are interleukins (IL)-4, 6, 7, 8, and 15, brain-derived neurotrophic factor (BDNF), irisin, and myostatin [15,17]. IL-15 regulates hypertrophy and myogenesis of muscle, suppresses lipid storage, and shows an antiobesity effect [15,18]. BDNF promotes lipid oxidation metabolism by the activation of AMP-activated protein kinase (AMPK) [15]. BDNF is also known as a major indicator of improvement in metabolic disorders and neuroplasticity through exercise [19]. Irisin causes white adipose tissue browning [20], and myostatin is involved in modulation of adipose tissue function and mass and maintenance of metabolic homeostasis [15].

Taekwondo is a popular combat sport worldwide among children and adolescents [21]. It has been suggested as a useful exercise program for physical education for students [22]. Several studies reported that Taekwondo training was effective for growth development, enhancing physical fitness, and improving body composition for growing subjects [21,23,24]. Kim et al. found that Taekwondo training was associated with positive improvement of body composition by inducing a decrease in body fat mass and percent body fat in adolescents [24]. This finding suggests that regular Taekwondo training has positive effects on oxidant–antioxidant balance and regulation of myokine secretion in overweight and obese adolescents. Nevertheless, there have been no studies of the effect of Taekwondo on relevant biomarkers in adolescents. Therefore, in the present study, we measured the effect of 16 weeks of regular Taekwondo training on oxidative stress and myokine levels in overweight and obese adolescents.

## 2. Methods

### 2.1. Participants

The participants were 20 overweight and obese adolescents (mean age ± standard deviation (SD), 12.55 ± 0.51 years; mean BMI ± SD, 24.33 ± 1.74 kg/m^2^; mean BMI z-score ± SD, 1.50 ± 0.31; mean BMI percentile ± SD, 92.42 ± 4.12%) who corresponded to the 85th percentile for BMI according to age and sex based on the 2007 Korean children and adolescent growth standards [25]. The number of subjects required was calculated to be 16 using G* Power software (G* Power 3.1.9.2, Heinrich-Heine-University, Düsseldorf, Germany) with effect size (ES) = 0.40, α value = 0.05, and desired statistical power (1-β) = 0.80. Finally, 20 subjects were selected given expected dropout. We randomly assigned them to the control group (CG) that underwent no treatment for 16 weeks and the experimental group (EG) who participated in Taekwondo training. Randomization was conducted using Microsoft Excel 2016 for Windows (Microsoft Inc., Redmond, WA, USA) as shown previously by Asghari and Sadeghi [26]. The characteristics of participants are shown in Table 1.

Selection criteria were as follows: (1) not participating in other regular exercise programs except for school physical education; (2) no experience of Taekwondo training; (3) no musculoskeletal disease; (4) not taking medications such as growth aids.

The purpose and procedure of this study were described to all subjects and their parents in detail, and each signed a research consent form that contained information on various matters and specifically informed them that they could stop participating in the study at will. The study protocol was approved by the ethics committee of the National Research Foundation of Korea (NRF-2015S1A5B5A02015262), and the study conformed to the standards set by the latest revision of the Declaration of Helsinki.

### 2.2. Research Procedure

We conducted subject selection, pretest, and post-test after the 16-week intervention. Parents of the subject were asked to record all foods through 24 h retrospective method that subject consumed for a day, and consumption of excessive calorie was restricted during experiment period based on past weekly record data [27]. Further, subjects were asked to not participate in any regular exercise program except the regular physical education class at school. At the pretest, we measured physical parameters (height, weight, and BMI), physical fitness (cardiorespiratory endurance, muscular strength, flexibility, power, and balance), and drew blood to examine blood oxidative stress biomarkers (MDA and SOD) and myokines (IL-15, BDNF, irisin, and myostatin). The EG performed 16 weeks of Taekwondo training, and the CG maintained daily life without any intervention for the same period. The post-test was conducted after the intervention in a manner similar to the pretest.

### 2.3. Measurement of Physical Fitness Variables

In the physical fitness measurement, we measured VO_2_ max (cardiorespiratory endurance), grip and leg strength (muscular strength), sit-and-reach (flexibility), Sargent jump (power), and stork stand test (balance). VO_2_ max was measured using the modified Balke treadmill protocol [28] on a treadmill (Q65, Quinton, CA, USA) while wearing a gas analyzer (MetaMax 3B, Cortex, Leipzig, Germany). Grip and leg strength were measured using a digital dynamometer (TKK 5401, Takei, Niigata, Japan) and leg muscle dynamometer (TKK 5402, Takei, Niigata, Japan) that measured in units of 0.1 kg. The better of two measurements was recorded. Sit-and-reach, Sargent jump, and stork stand test were measured using physical fitness measurement system (Helmas-III, O_2_run, Seoul, Korea) as described in “Advanced Fitness Assessment and Exercise Prescription” [29]; the better of two measurements was recorded.

### 2.4. Blood Collection and Analysis

After an 8 h fast, 10 mL blood was drawn at rest from an antecubital vein using a 22-gauge needle, serum separator tube, and ethylenediamine tetra-acetic acid tube. Samples were centrifuged for 15 min at 3000 rpm and stored at –80 °C until analysis. Analysis of plasma MDA levels and SOD activities was conducted using colorimetric assay as previously described [30]. Plasma MDA levels were determined using a commercial kit (#21012, Oxis International, Portland, OR, USA). A 200-μl aliquot of plasma or standard was mixed with 640 μl of diluted N-methyl-2-phenylindole. Then, 150 μl of concentrated hydrochloric acid was added, mixed, and incubated at 45 °C (60 min). After cooling, the absorbance values of the standards and samples were read at 586 nm using a spectrophotometer (HP 8452A, Hewlett-Packard, Palo Alto, CA, USA). Plasma SOD activities were determined using a tetrazolium-based kit (#CM706002, IBL International, Germany). A 200 μl sample of the diluted radical detector and 10 μl plasma sample were added to prepared standard wells. Then, 20 μl of diluted xanthine oxidase was added and mixed for a few seconds. The reaction was incubated at room temperature. After incubation, the absorbance values of the standards and samples were read at 450 nm using a spectrophotometer (Tecan Sunrise, TECAN GmbH, Salzburg, Austria). Serum IL-15 (#D1500, R&D Systems, Minneapolis, MN, USA), BDNF (#DY248, R&D Systems, Minneapolis, MN, USA), irisin (#EK-067-52, Phoenix Pharmaceuticals, Belmont, CA, USA), and myostatin (#DY788-05, R&D Systems, Minneapolis, MN, USA) levels were analyzed by enzyme-linked immunosorbent assay (ELISA). To be specific, a 100 µl sample was divided into each well and attached to the plate using adhesive strips; this was left to react for 2 h at room temperature. Then, 100 µl detection antibody was divided among the wells after washing three times and it was incubated for 2 h at room temperature after attaching it with an adhesive strip. Then, 100 µl of streptavidin-HRP was divided into each well after washing three times, and it was incubated for 2 h at room temperature without direct sunlight after covering the wells. Further, 100 µl of substrate solution was divided into each well after washing three times for 20 min at room temperature without direct sunlight after covering the wells. Finally, 50 µl stop solution was added to each well, and the product was analyzed at 450 nm using a spectrophotometer (Tecan Sunrise, TECAN GmbH, Salzburg, Austria).

### 2.5. Taekwondo Training Intervention

Taekwondo training was conducted for 60 minutes, five times a week, for 16 weeks according to the Taekwondo intervention method by Cho et al. [31]. All interventions were conducted through demonstration and instruction by a Taekwondo expert. Warm-up, main exercise, and cool-down were conducted in order. Details of the Taekwondo training program are shown in Table 2.

### 2.6. Statistical Analysis

Data were expressed as mean ± standard deviation for all dependent variables. Tests of normality for all measured values were conducted using the one-sample Kolmogorov–Smirnov test. Analysis was performed using SPSS version 25.0 for Windows (IBM Corp., Armonk, NY, USA). Two-way repeated measures analysis of variance (ANOVA) that measured time repetitively was performed to examine the time and group differences of each dependent variable. In case of statistically significant interaction effects, independent *t*-tests for differences between two groups within time and paired *t*-tests for differences before and after exercise within group were conducted. All statistical significance levels (α) were set at 0.05.

## 3. Results

### 3.1. Change in Physique and Physical Fitness

The changes in physique and physical fitness in the CG and EG before and after 16 weeks intervention are shown in Table 3. Weight (F = 22.446, *p <* 0.001) and BMI (F = 23.993, *p <* 0.001) showed significant interactions (time X group) between groups. The CG showed no significant differences before and after intervention (*p >* 0.05), whereas the EG showed significantly lower weight and BMI than before the intervention (*p* < 0.05). Leg strength (F = 5.294, *p =* 0.034), sit-and-reach (F = 4.803, *p =* 0.042), and Sargent jump (F = 5.504, *p =* 0.031) showed significant interactions (time X group) between groups. The CG showed no significant difference before and after intervention (*p >* 0.05), whereas the EG showed significantly increased leg strength, sit-and-reach, and Sargent jump records that represent muscular strength, flexibility, and power, respectively, after the intervention (*p <* 0.05). There was no significant interaction effects in height, VO_2_ max, grip strength, and stork stand test (*p >* 0.05).

### 3.2. Changes in Oxidative Stress Biomarkers

The changes in oxidative stress biomarker levels before and after 16 weeks intervention in the CG and EG are shown in Table 4. Both MDA (F = 18.904, *p <* 0.001) and SOD (F = 15.193, *p =* 0.001) showed significant interactions (time X group) between groups. The CG showed no significant differences (*p >* 0.05), whereas the EG showed significantly lower serum levels of MDA and significantly higher serum SOD activities than before the intervention (*p <* 0.05).

### 3.3. Changes in Myokine Levels

Changes in myokine levels before and after the 16-week intervention are shown in Table 5. BDNF (F = 6.767, *p =* 0.018) and irisin (F = 8.566, *p =* 0.009) showed significant interactions (time X group) between groups. The CG showed no significant differences before and after the intervention (*p >* 0.05), whereas the EG showed significantly greater serum BDNF levels and significantly lower serum irisin levels than before intervention (*p <* 0.05). There were no significant interaction effects in serum IL-15 and myostatin levels (*p >* 0.05).

## 4. Discussion

This study investigated the effects of Taekwondo training in overweight and obese adolescents and demonstrated beneficial effects in terms of oxidative stress alleviation and regulation of myokine secretion. To the best of our knowledge, ours is the first study of its kind to examine changes in oxidative stress levels and myokine secretion associated with Taekwondo training in overweight and obese adolescents.

About 80 million people over 180 countries participate in Taekwondo, a Korean martial art that is popular with children and adolescents [21,32]. It was reported that Taekwondo training is effective not only for improving physical health but also for emotional and social development, including improvement of cognitive function and social-emotional conditioning in growing subjects [22,24,32].

We measured height, weight, BMI, and recorded VO_2_ max, grip strength, leg strength, sit-and-reach, Sargent jump, and stork stand test results to examine the effect of Taekwondo training on physique and physical fitness. As a result of Taekwondo training, weight and BMI were significantly reduced while leg strength, sit-and-reach, and Sargent jump measurements were significantly improved. These results suggest that Taekwondo training can reduce obesity in adolescents and can improve lower limb strength, flexibility, and power. The data also support the findings of studies showing that Taekwondo training is effective in improving body composition and physical fitness. These results may be attributable to repetitively performed Taekwondo motions in the intervention program, including static and ballistic stretching and various stance motions that stimulate weight-bearing muscles such as the leg and hip as well as frequent jumping and weight shifting kicking. Kim et al. reported that Taekwondo training 50 min once and twice a week for 12 weeks in adolescents produced no changes in cardiorespiratory endurance or grip strength; however, there was significant improvements in standing long jump (representing muscle power) and sit-and-reach (representing isokinetic knee muscle strength and flexibility) as well as significant decreases in percent body fat and fat body mass [24]. Similar to our results, Fong and Ng found that regular Taekwondo training can improve body composition through fat loss and flexibility [33].

Obesity can induce systemic oxidative stress by various biochemical mechanisms such as superoxide generation from NADPH oxidases, oxidative phosphorylation, glyceraldehyde auto-oxidation, protein kinase C activation, and polyol and hexosamine pathways [8]. Oxidative stress sensitively reacts via ROS and induces lipid peroxidation by deformation of polyunsaturated fatty acids, which are important components of cell membranes; the marker that represents this condition is MDA [34]. On the other hand, the body has an efficient antioxidant defense system to protect from oxidative stress, consisting of antioxidant enzymes such SOD, catalase, and GPx [35]. Regular exercise training can increase antioxidant capacity [36,37]. In the present study, we measured plasma MDA levels and SOD activities to examine the effect of Taekwondo training on oxidative stress in overweight and obese adolescents. We found that plasma MDA levels were significantly lower and plasma SOD activity was significantly greater after Taekwondo training. This suggests that Taekwondo training alleviates oxidative stress in overweight and obese adolescents. Taekwondo training may have beneficial effects similar to those of endurance- and resistance-type exercises.

Azizbeigi et al. measured serum levels of MDA, SOD, GPx, and total antioxidant capacity (TAC) after eight weeks of endurance, resistance, and combined training to examine the effect of exercise type on circulating antioxidant capacity and oxidative stress. They found that all types of training were associated with lower MDA levels and increased SOD activity [38]. It is possible that the independent effects of regular exercise and alleviation of obesity induced alleviation of oxidative stress. Shih et al. reported significantly increased SOD activity after weight loss in overweight and obese subjects [39]. Li et al. showed markedly improved activities of antioxidant enzymes such as SOD and GPx by improving obesity in obese adolescents using exercise and dietary restriction [14], supporting the result of this study.

Myokines are active substances that are synthesized in the skeletal muscle and regulate the function of tissues such as fat, liver, pancreas, bone, brain, and muscle using autocrine, paracrine, or endocrine mechanisms [15]. Exercise can stimulate the secretion of myokines that protect functionality and enhance the exercise capacity of the skeletal muscle [40]. Myokines act as paracrine regulators of energy oxidation, hypertrophy, angiogenesis, inflammatory processes, and regulation of the extracellular matrix as well as regulate adaptive processes of the skeletal muscle [40]. They also have reported endocrine functions in body weight regulation, low-grade inflammation, insulin sensitivity, suppression of tumor growth, and improvement of cognitive function [40].

In the present study, we measured serum levels of IL-15, BDNF, irisin, and myostatin to study the effect of Taekwondo training on myokine secretion in overweight and obese adolescents. We found no significant difference in serum IL-15 and myostatin levels after Taekwondo training; however, BDNF levels were significantly greater and irisin levels were significantly lower. This result suggests that Taekwondo training modulates myokine secretion.

BDNF is involved in AMPK-mediated fat oxidation, myogenesis, satellite cell activation, and skeletal muscle regeneration. It plays an important role in the interaction between the skeletal muscle and the brain [15,41]. Based on evidence that serum BDNF levels were lower in patients with type 2 diabetes and obesity [13,41,42], one can speculate that serum BDNF levels increased as Taekwondo training alleviated obesity in overweight and obese adolescents.

Irisin is a small peptide (12 kDa) that is formed by proteolysis of fibronectin III domain-containing protein 5 (FNDC5). It is a membrane-bound protein secreted from skeletal muscle in reaction to physical activity or shivering [16]. Exercise was shown to induce irisin secretion [20,41]. Nevertheless, some studies did not demonstrate the activation of the FNDC5 gene due to exercise [43,44,45]; rather, human studies reported that regular exercise had an inverse correlation with circulating irisin levels [45,46]. The present study showed significant decrease in serum irisin levels after Taekwondo training. This may be related to the decrease in BMI even though it is difficult to suggest a specific mechanism. Palacios-González et al. examined serum irisin levels according to BMI in school-age children, and found a positive correlation between BMI and serum irisin levels; obese children showed higher serum irisin levels than normal weight and overweight children [47]. Jang et al. analyzed serum irisin level in Korean adolescents and found that obese adolescents showed significantly higher serum irisin levels than did normal weight adolescents, supporting the results of the present study [48].

The strength of this study is that it is the first of its kind to examine changes in oxidative stress levels and myokine secretion associated with Taekwondo training in overweight and obese adolescents. Nevertheless, there are some limitations. First, the subjects were recruited in Seoul, Korea; therefore, the sample may not represent the whole Korean population of adolescents with obesity. Second, the number of subjects was small and sex differences were not considered. Nevertheless, we were able to generate statistically significant results. A future study should consider a larger number of subjects as well as sex differences. Third, this was a single-center study, and thus, selection bias cannot be excluded. However, we introduced strict exclusion criteria in an attempt to mitigate this bias. Fourth, daily calorie intake and components of nutrition were not analyzed. Future studies should examine this using a computer-aided nutrition program. Finally, the study was restricted to changes in obesity and we did not measure body composition in detail. For this reason, future studies should include measurements of skeletal muscle mass, body fat mass, and percent body fat using bioelectrical impedance analysis and/or dual X-ray absorptiometry.

## 5. Conclusions

Taekwondo training can reduce obesity and increase physical fitness in the form of muscular strength, flexibility, and power as well as alleviate oxidative stress and modulate myokine secretion in adolescents.

## Figures and Tables

**Table 1:** Characteristics of the participants at baseline.

Group Variables	CG	EG	*p*
Number (boys/girls)	10 (7/3)	10 (7/3)	
Age (years)	12.50 ± 0.53	12.60 ± 0.52	0.673
School grades (unit)	5.50 ± 0.53	5.60 ± 0.52	0.673
Height (cm)	151.96 ± 6.87	153.07 ± 5.78	0.700
Weight (kg)	54.99 ± 7.06	58.34 ± 7.13	0.305
BMI (kg/m^2^)	23.74 ± 1.43	24.91 ± 1.90	0.137
BMI z-score	1.44 ± 0.28	1.56 ± 0.33	0.398
BMI percentile (%)	91.60 ± 4.31	93.24 ± 3.97	0.388
VO_2_ max (ml/kg/min)	35.42 ± 5.20	36.64 ± 5.38	0.612
Grip strength (kg)	20.49 ± 3.41	20.58 ± 3.47	0.954
Leg strength (kg)	46.24 ± 9.70	46.85 ± 7.64	0.878
Sit-and-reach (cm)	6.11 ± 6.87	6.09 ± 8.35	0.995
Sargent jump (cm)	28.70 ± 6.55	28.60 ± 6.13	0.972
Stork stand test (sec)	42.40 ± 34.40	39.70 ± 33.38	0.861
MDA (nmol/ml)	5.20 ± 0.91	5.41 ± 1.40	0.697
SOD (U/ml)	3.59 ± 0.62	3.34 ± 0.45	0.303
IL-15 (pg/ml)	2.34 ± 0.58	2.25 ± 0.95	0.801
BDNF (ng/ml)	26.58 ± 6.10	25.41 ± 5.36	0.654
Irisin (ng/ml)	143.09 ± 30.17	145.81 ± 32.18	0.848
Myostatin (ng/ml)	1.65 ± 0.43	1.70 ± 0.52	0.825

Data are presented as mean ± standard deviation. CG, control group; EG, experimental group; BMI, body mass index; VO_2_ max, maximal oxygen uptake; MDA, malondialdehyde; SOD, superoxide dismutase; IL-15, interleukin-15; BDNF, brain-derived neurotrophic factor. *p*-value as determined using the independent *t*-test for each of the two groups at baseline.

**Table 2:** Taekwondo training programs.

Constitution		Main Activity
Warm-up (5 min)		Stretching
Main Exercise	Basal fitness training (10 min)	Push-up, Sit-up, Shuttle-run, Burpee test, Vertical jump, Jumping over a person
	Basic motion of Taekwondo (5 min)	Close stance, Parallel stance, Riding stance, Forward stance, Forward inflection stance, Backward inflection stance, Body punch in horse-riding stance
	Poomsae (10 min)	Taegeuk chapter 1–8
	Kicking (10 min)	Kick (Front, Side, Round house, Downward), Step (Forward, Side, Backward), Practice mitt kicking
	Taekwon gymnastics (15 min)	Gymnastics of Taekwondo motion to music
Cool-down (5 min)		Stretching

**Table 3:** Change in physique and fitness over the 16-week intervention.

Group Variables	CG		EG		Time × Group Interaction	
	Pre	Post	Pre	Post	F	*p*
Height (cm)	151.96 ± 6.87	152.25 ± 6.77	153.07 ± 5.78	153.83 ± 5.97	2.551	0.128
Weight (kg)	54.99 ± 7.06	55.34 ± 6.51	58.34 ± 7.13	55.99 ± 6.53 †	22.446	< 0.001 ***
BMI (kg/m^2^)	23.74 ± 1.43	23.82 ± 1.42	24.91 ± 1.90	23.59 ± 1.84 †	23.993	< 0.001 ***
VO_2_ max (ml/kg/min)	35.42 ± 5.20	35.45 ± 5.66	36.64 ± 5.38	36.46 ± 5.76	0.064	0.804
Grip strength (kg)	20.49 ± 3.41	20.93 ± 3.47	20.58 ± 3.47	21.08 ± 3.31	0.010	0.920
Leg strength (kg)	46.24 ± 9.70	46.54 ± 9.51	46.85 ± 7.64	48.79 ± 9.30 †	5.294	0.034 *
Sit-and-reach (cm)	6.11 ± 6.87	6.15 ± 6.82	6.09 ± 8.35	7.48 ± 6.94 †	4.803	0.042 *
Sargent jump (cm)	28.70 ± 6.55	29.60 ± 8.15	28.60 ± 6.13	31.80 ± 6.30 †	5.504	0.031 *
Stork stand test (sec)	42.40 ± 34.40	43.00 ± 31.53	39.70 ± 33.38	39.80 ± 23.65	0.015	0.905

Data are presented as mean ± standard deviation. CG, control group; EG, experimental group; BMI, body mass index; VO_2_ max, maximal oxygen uptake; †, Significant difference pre- and post-intervention within the group (*p* < 0.05); *** *p* < 0.001; * *p* < 0.05.

**Table 4:** Change in oxidative stress biomarkers over the 16-week intervention.

Group Variables	CG		EG		Time × Group Interaction	
	Pre	Post	Pre	Post	F	*p*
MDA (nmol/ml)	5.20 ± 0.91	5.32 ± 0.93	5.41 ± 1.40	4.79 ± 1.21 †	18.904	<0.001 ***
SOD (U/ml)	3.59 ± 0.62	3.51 ± 0.57	3.34 ± 0.45	3.46 ± 0.48 †	15.193	0.001 **

Data are presented as mean ± standard deviation. CG, control group; EG, experimental group; MDA, malondialdehyde; SOD, superoxide dismutase; † Significant difference pre- and post-intervention within the group (*p* < 0.05); *** *p* < 0.001; ** *p* < 0.01.

**Table 5:** Change in myokines over the 16-week intervention.

Group Variables	CG		EG		Time × Group Interaction	
	Pre	Post	Pre	Post	F	*p*
IL-15 (pg/ml)	2.34 ± 0.58	2.50 ± 0.56	2.25 ± 0.95	2.30 ± 0.78	0.344	0.565
BDNF (ng/ml)	26.58 ± 6.10	27.68 ± 6.50	25.41 ± 5.36	29.52 ± 5.83 †	6.767	0.018 *
Irisin (ng/ml)	143.09 ± 30.17	142.78 ± 30.71	145.81 ± 32.18	136.09 ± 28.22 †	8.566	0.009 **
Myostatin (ng/ml)	1.65 ± 0.43	1.63 ± 0.45	1.70 ± 0.52	1.75 ± 0.35	0.400	0.535

Data are presented as mean ± standard deviation. CG, control group; EG, experimental group; IL-15, interleukin-15; BDNF, brain-derived neurotrophic factor; † Significant difference pre- and post-intervention within the group (*p* < 0.05); ** *p* < 0.01; * *p* < 0.05.
